# Non-linear VLBI station motions and their impact on the celestial reference frame and Earth orientation parameters

**DOI:** 10.1007/s00190-015-0830-4

**Published:** 2015-06-18

**Authors:** Hana Krásná, Zinovy Malkin, Johannes Böhm

**Affiliations:** Department of Geodesy and Geoinformation E120/4, Vienna University of Technology, Gußhausstraße 27-29, 1040 Vienna, Austria; Pulkovo Observatory, St. Petersburg, 196140 Russia; St. Petersburg State University, St. Petersburg, 199034 Russia; Kazan Federal University, Kazan, 420008 Russia

**Keywords:** Surface deformation, VLBI, GRACE, Celestial reference frame, Earth orientation parameters

## Abstract

The increasing accuracy and growing time span of Very Long Baseline Interferometry (VLBI) observations allow the determination of seasonal signals in station positions which still remain unmodelled in conventional analysis approaches. In this study we focus on the impact of the neglected seasonal signals in the station displacement on the celestial reference frame and Earth orientation parameters. We estimate empirical harmonic models for selected stations within a global solution of all suitable VLBI sessions and create mean annual models by stacking yearly time series of station positions which are then entered a priori in the analysis of VLBI observations. Our results reveal that there is no systematic propagation of the seasonal signal into the orientation of celestial reference frame but position changes occur for radio sources observed non-evenly over the year. On the other hand, the omitted seasonal harmonic signal in horizontal station coordinates propagates directly into the Earth rotation parameters causing differences of several tens of microarcseconds.

## Introduction

Realizations of Terrestrial Reference Frames (TRF), such as the International Terrestrial Reference Frame 2008 (ITRF 2008, Altamimi et al. 2011) and the Very Long Baseline Interferometry (VLBI) Terrestrial Reference Frame 2008 (VTRF 2008, Böckmann et al. 2010), define station positions as the sum of the coordinates at a certain time epoch plus a linear velocity term times the time span elapsed since the reference epoch. In the analysis of space geodetic techniques several tidal corrections, e.g. the solid Earth tides, the tidal ocean and tidal atmospheric loading displacement are added as recommended in the Conventions 2010 of the International Earth Rotation and Reference Systems Service (IERS) (Petit and Luzum 2010). In the analysis of VLBI observations, corrections for non-tidal atmospheric loading are normally applied as well.

However, seasonal signals with amplitudes of several millimetres are still present in most of the station position time series, as shown by several authors for VLBI and the Global Positioning System (GPS) position time series, cf. Titov and Yakovleva (2000), Blewitt et al. (2001), van Dam et al. (2001, 2012), Dong et al. (2002), Petrov and Ma (2003), Ding et al. (2005), Collilieux et al. (2007), Tesmer et al. (2009, 2011) or Eriksson and MacMillan (2014). They conclude that parts of the remaining seasonal signal have a geophysical origin, mainly from hydrology and—to a lesser extent—from non-tidal ocean loading. The impact of seasonal station motion on Universal Time (UT1) from Intensive sessions (VLBI sessions with 1 h duration and maximal two baselines) was investigated by Malkin (2013). The effect of loading displacement on the seasonal variations of the GPS frame origin and orientation, in particular based on the Gravity Recovery and Climate Experiment (GRACE) model, was also performed in several studies, e.g., by Collilieux et al. (2012) or Zou et al. (2014). They focused mainly on the aliasing of the seasonal variations in station positions into the terrestrial reference frame transformation parameters.

In this paper we investigate the propagation of the seasonal signals in station coordinates into the Celestial Reference Frame (CRF) and Earth Orientation Parameters (EOP) estimated from 24-h VLBI sessions. After defining the setup of our VLBI analysis in Sect. 2 we introduce the harmonic and mean annual station models in Sect. 3. In Sect. 4 we compare the models with loading series derived from hydrology and GRACE before we assess the impact of neglected station motions on CRF and EOP.

## Analysis setup and global reference frames

For our investigation we reprocessed a long time span of VLBI data from about 3700 24-h sessions of the International VLBI Service for Geodesy and Astrometry (IVS, Schuh and Behrend 2012) from 1984.0 until 2013.3. The processing was done with the Vienna VLBI Software VieVS (Böhm et al. 2012) using state-of-the-art models following the IERS Conventions 2010. We used the ocean tidal loading corrections based on the FES2004 model (Lyard et al. 2006) provided by Bos and Scherneck^1^ and the non-tidal atmospheric pressure loading time series by the Goddard VLBI group (Petrov and Boy , 2004).^2^ Pole tide and ocean pole tide were corrected with the cubic approximation of the mean pole model, the thermal deformation was modelled according to Nothnagel (2009), and the tropospheric delays under various elevation angles were mapped into the zenith direction with the Vienna mapping functions VMF1 (Böhm et al. 2006). We used the concept of piece-wise linear offsets for the parameterisation of the station-dependent clock parameters, zenith wet delays, and troposphere gradients, see Table 1 for the interval lengths and constraints. The Earth orientation parameters (polar motion, UT1, celestial pole offsets) were estimated as single offsets for the whole 24-h session. In the global adjustment of all sessions the terrestrial and celestial reference frames were estimated in one common least-squares adjustment. For more details about VLBI analysis we refer to Schuh and Böhm (2013).

**Table 1:** Interval lengths between the piece-wise linear offsets of auxiliary parameters and relative constraints added as pseudo-observations to the Jacobian matrix of the least-squares adjustment

Parameter	Interval (min)	Relative constraints
Clock	60	1.3 cm after 60 min
zwd	60	1.5 cm after 60 min
Trop. gradients	360	0.05 cm after 360 min

The TRF (called VieTRF13b) contains coordinates and linear velocities of 66 telescopes estimated as global parameters and mean coordinate offsets of session-wise estimated coordinates of 36 telescopes which were reduced from the normal equation system with fixed velocity because of the poor data span of observations not allowing for a reliable velocity determination. The datum was defined with no-net-translation (NNT) and no-net-rotation (NNR) conditions with respect to the VTRF2008 applied at 22 stations with a long observation history. Table 2 shows the seven Helmert parameters for transformation between the VieTRF13b and the VTRF2008 at epoch 2000.0. The coordinates and velocities were weighted according to the formal errors derived in the VieTRF13b solution. The second column shows the parameters between stations with mean coordinate errors $m_{xyz}$ lower than 5 mm and the third column Helmert parameters for all globally estimated stations. Except of $T_x$ ($2.53~\pm$ 0.82 mm) and $R_z$ ($53~\pm$ 26 $\upmu$as) all parameters agree with zero within their formal errors.

**Table 2:** Weighted Helmert parameters between VieTRF13b and VTRF2008

Parameter	$m_{xyz}<$ 5 mm	All stations
$T_x$ (mm)	$2.40~\pm$ 0.69	$2.53~\pm$ 0.82
$T_y$ (mm)	$-0.95~\pm$ 0.71	$-0.88~\pm$ 0.84
$T_z$ (mm)	$0.04~\pm$ 0.66	$-0.07~\pm$ 0.79
$R_x$ ($\upmu$as)	$16~\pm$ 27	$16~\pm$ 32
$R_y$ ($\upmu$as)	$25~\pm$ 27	$27~\pm$ 31
$R_z$ ($\upmu$as)	$53~\pm$ 22	$53~\pm$ 26
Scale (ppb)	$0.02~\pm$ 0.10	$-0.02~\pm$ 0.12

The mean coordinate error was computed as: $m_{xyz} = \sqrt{(m_{x}^2 + m_{y}^2 + m_{z}^2)/3}$ where $m_{x}^2, m_{y}^2,$ and $m_{z}^2$ are variances of the respective coordinates

The CRF (called VieCRF13b) consists of coordinates of 871 globally adjusted radio sources and mean offsets of 39 so-called special handling sources which were session-wise reduced from the normal equations due to their apparent position changes. We did not include Very Long Baseline Array (VLBA) Calibrator Survey sessions in our analysis. The alignment with the ICRF2 (Ma et al. 2009) catalogue was evaluated via rotation on 285 ICRF2 defining sources which were observed more than 20 times in our dataset. The weighted rotation parameters computed between radio sources with a mean coordinate error $m_\mathrm{RADe}$ lower than 1 mas are listed in the second column of Table 3 and those between all sources are shown in the third column. All of them agree with zero within their formal errors.

**Table 3:** Weighted rotation parameters between VieCRF13b and ICRF2

Parameter	$m_{\mathrm{RADe}}<$ 1 mas	All sources
A1 ($\upmu$as)	$0.01~\pm$ 0.68	$-0.25~\pm$ 1.12
A2 ($\upmu$as)	$0.04~\pm$ 0.68	$-0.09~\pm$ 1.16
A3 ($\upmu$as)	$-0.06~\pm$ 0.65	$-0.02~\pm$ 0.84

The mean coordinate error was computed as: $m_{\mathrm{RADe}} = \sqrt{(m_{\mathrm{RA}}^2 + m_{\mathrm{De}}^2)/2}$ where $m_{\mathrm{RA}}^2$ and $m_{\mathrm{De}}^2$ are variances of the respective coordinates

## Empirical models for seasonal station motion

Seasonal station displacement models were developed for all stations participating in more than 50 sessions and with observations evenly distributed over all months to avoid singularity in the least-squares adjustment. For example, we excluded station O’Higgins in Antarctica where measurements are only collected during Antarctic summer months. For the derivation of the model, we basically follow the same parameterisation as described in Sect. 2 and use VieTRF13b and VieCRF13b as reference frames.

### Harmonic model of station displacements

The first model is a harmonic model for station displacements at annual ($P = 365.25$ days) and semi-annual periods ($P = 182.625$ days). The signal was estimated in form of sine and cosine amplitudes at the chosen periods as additional parameters in the adjustment together with terrestrial and celestial reference frames in the global solution of the VLBI sessions. Equation (1) shows the relation between the topocentric (height, east, north) station displacement $\Delta d_{\mathrm{HEN}}$ and the estimated sine ($A_\mathrm{s}$) and cosine ($A_\mathrm{c}$) amplitudes of the deformation:

1 $$\begin{aligned} \Delta d_{\mathrm{HEN}}= & {} A_\mathrm{c_{\mathrm{HEN}}} \cdot \cos \bigg (\frac{(\mathrm{mjd}-\mathrm{mjd}_0)}{P}2\pi \bigg ) \nonumber \\&+\, A_\mathrm{s_{\mathrm{HEN}}} \cdot \sin \bigg (\frac{(\mathrm{mjd}-\mathrm{mjd}_0)}{P}2\pi \bigg ), \end{aligned}$$

where *P* is the period in solar days, the modified Julian date of the reference time epoch mjd$_0$ is set to J2000.0, and mjd stands for the modified Julian date of the observation. The components of the amplitude $A_{\mathrm{HEN}}$ in the local system with the corresponding phase $\phi _{\mathrm{HEN}}$ are obtained as

2 $$\begin{aligned}&A_{\mathrm{HEN}} = \sqrt{A_\mathrm{c_{\mathrm{HEN}}}^2 + A_\mathrm{s_{\mathrm{HEN}}}^2},\end{aligned}$$

3 $$\begin{aligned}&\phi _{\mathrm{HEN}} = \arctan {\bigg (\frac{A_\mathrm{s_{\mathrm{HEN}}}}{A_\mathrm{c_{\mathrm{HEN}}}} \bigg )}. \end{aligned}$$

**Fig. 1:**
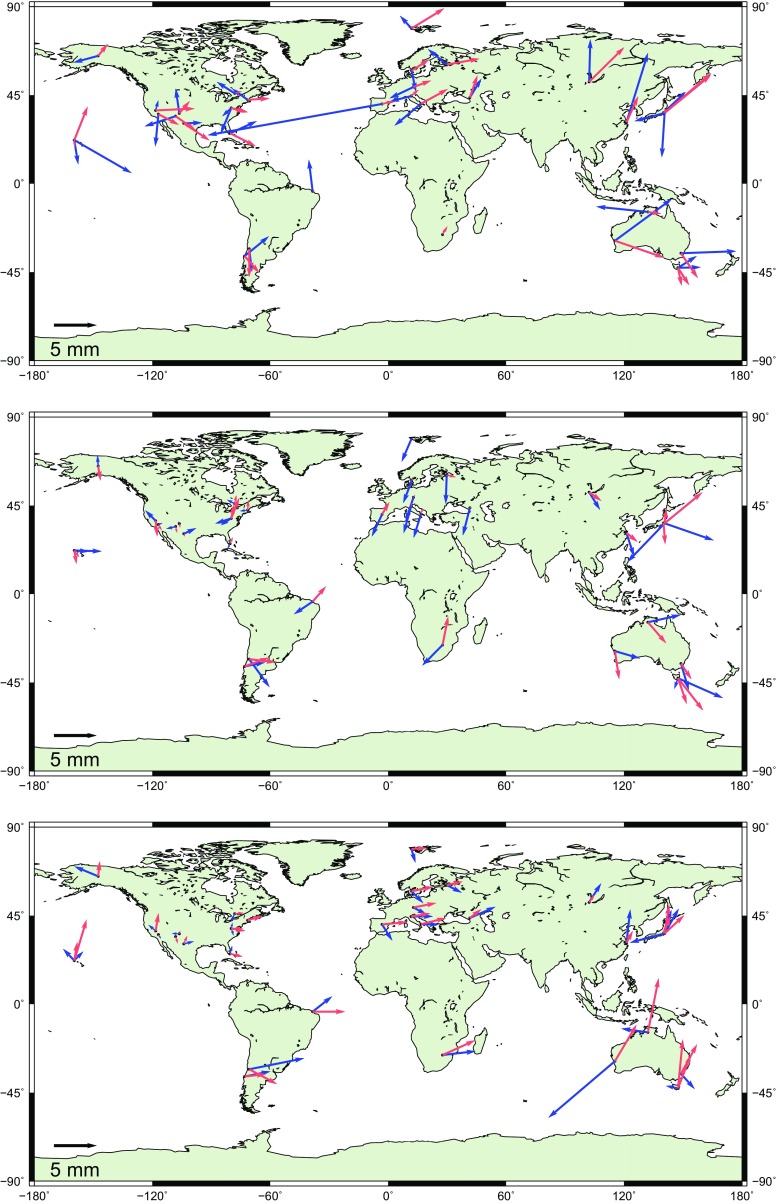
Amplitudes of annual (*blue*) and semi-annual (*light red*) harmonic signals in height (*upper plot*), east (*middle plot*) and north (*lower plot*) direction at stations participating in more than 50 sessions. The *length of the arrow* depends on the estimated amplitude and the direction depicts the month of the maximum displacement starting in the north direction for January continuing clock-wise

The upper plot in Fig. 1 shows the estimated height amplitudes of the annual (blue) and semi-annual (light red) harmonic signal. The length of the arrow corresponds to the estimated amplitude and the direction depicts the month of the maximum displacement starting in north direction for January and continuing clock-wise. The mean value over all estimated annual amplitudes is 3.6 mm, not considering station Yebes 40 m with an annual amplitude of 21 $\pm$ 1 mm where the amplitude estimation of the seasonal movement in global adjustment is not reliable due to the relative short observation time of only three years. Visual comparison with harmonic annual signals at 17 sites presented by Tesmer et al. (2009) in their Fig. 3 shows a similar pattern with our harmonic signals in terms of the amplitudes and the phases. The mean value of the estimated semi-annual amplitudes in height is 2.9 mm. The phase of the semi-annual signal is similar at most stations within a certain global region, confirming the geophysical nature of the signal. In Europe the maximal displacement in the semi-annual signal occurs in February and August, in North America in April and October.

The middle and lower plots in Fig. 1 depict the east and the north amplitudes of the seasonal displacement in the horizontal plane. At most of the stations, the size of the horizontal amplitudes is comparable with the amplitudes in height. The estimated phases of the displacement are similar at stations placed in the same area. For example in Europe, the east components of the annual displacements have their maxima at around July at all stations, whereas the semi-annual components nearly vanish (a mean value of 0.6 mm). The annual displacements in north direction in Europe are largest in spring and the semi-annual displacements reach their maxima in March and September. In North America the estimated seasonal displacements in the horizontal plane are weaker (1.3 and 1.1 mm in east and north direction for the annual amplitudes, and 1.2 and 1.3 mm for the semi-annual amplitudes) in comparison to the rest of the stations. The mean value over all stations for the estimated annual amplitudes is 2.5 mm in the east direction and 2.4 mm in the north direction. For the semi-annual signal the mean values are 1.8 and 2.6 mm for east and north direction, respectively. Ding et al. (2005) or Malkin (2013) found unmodelled semi-annual displacement in the horizontal plane with values generally up to 1.0 mm. The origin of the estimated semi-annual signal is partly of real physical nature, e.g., due to mismodelling the geophysical factors having impact on the station displacement, such as the atmospheric loading and tides, and partly due to the fact that annual harmonics cannot account for the seasonal variations appropriately. Table 4 summarizes the estimated parameters of the harmonic model for the ten most frequently observed stations. The first lines contain the height, east and north components of the amplitudes and phases of the annual displacement, followed by the second lines describing the estimated semi-annual components.

**Table 4:** Amplitudes and phases (HEN) of the harmonic model at annual (first lines) and semi-annual (second lines) periods at ten most frequently observed stations

Station	$A_\mathrm{H}$ (mm)	$\phi _{\mathrm{H}}$ ($\circ$)	$A_\mathrm{E}$ (mm)	$\phi _{\mathrm{E}}$ ($\circ$)	$A_\mathrm{N}$ (mm)	$\phi _{\mathrm{N}}$ ($\circ$)
ALGOPARK	$2.5~\pm$ 0.7	$303~\pm$ 16	$1.0~\pm$ 0.3	$297~\pm$ 15	$1.0~\pm$ 0.3	$203~\pm$ 17
	$0.8~\pm$ 0.7	$153~\pm$ 45	$1.0~\pm$ 0.3	$23~\pm$ 15	$1.1~\pm$ 0.3	$170\pm 14$
FORTLEZA	$3.8~\pm$ 0.8	$354~\pm$ 11	$2.6~\pm$ 0.6	$236~\pm$ 12	$2.9~\pm$ 0.6	$52\pm 12$
	$0.4~\pm$ 0.7	$227~\pm$ 117	$2.4~\pm$ 0.5	$83~\pm$ 14	$3.8~\pm$ 0.6	$180\pm 8$
GILCREEK	$3.0~\pm$ 0.4	$255~\pm$ 8	$1.3~\pm$ 0.3	$356~\pm$ 12	$3.0~\pm$ 0.3	$293\pm 5$
	$1.7~\pm$ 0.4	$77~\pm$ 14	$1.7~\pm$ 0.3	$342~\pm$ 8	$1.8~\pm$ 0.3	$12\pm 8$
HARTRAO	$0.6~\pm$ 1.0	$25~\pm$ 97	$3.4~\pm$ 0.9	$224~\pm$ 15	$3.9~\pm$ 0.8	$83\pm 15$
	$1.1\pm 0.9$	$58~\pm$ 49	$3.4~\pm$ 0.9	$22~\pm$ 13	$4.2~\pm$ 0.9	$131\pm 12$
KOKEE	$2.9\pm 0.6$	$171~\pm$ 10	$1.7~\pm$ 0.4	$94~\pm$ 12	$1.9~\pm$ 0.4	$316\pm 13$
	$0.9~\pm$ 0.5	$353~\pm$ 33	$1.0~\pm$ 0.3	$286~\pm$ 22	$2.2~\pm$ 0.4	$19\pm 10$
MATERA	$4.0~\pm$ 0.6	$231~\pm$ 9	$2.9~\pm$ 0.5	$197~\pm$ 8	$2.1~\pm$ 0.4	$91\pm 14$
	$3.6~\pm$ 0.6	$123~\pm$ 10	$0.7~\pm$ 0.4	$35~\pm$ 34	$3.0~\pm$ 0.4	$152\pm 8$
NYALES20	$2.0~\pm$ 0.4	$317~\pm$ 12	$2.9~\pm$ 0.3	$203~\pm$ 6	$1.7~\pm$ 0.3	$164\pm 10$
	$4.5~\pm$ 0.4	$117~\pm$ 5	$0.2~\pm$ 0.3	$145~\pm$ 70	$1.8~\pm$ 0.3	$173\pm 9$
TIGOCONC	$3.6~\pm$1.0	$52~\pm$ 17	$2.4~\pm$ 0.7	$79~\pm$ 19	$3.1~\pm$ 0.8	$79\pm 15$
	$2.6~\pm$1.0	$279~\pm$ 23	$3.0~\pm$ 0.7	$140~\pm$ 14	$2.5~\pm$ 0.8	$167\pm 16$
WESTFORD	$2.2~\pm$ 0.4	$306~\pm$ 11	$0.9~\pm$ 0.2	$264~\pm$ 15	$0.4~\pm$ 0.3	$45\pm 35$
	$2.6~\pm$ 0.4	$172~\pm$ 9	$1.2~\pm$ 0.2	$10~\pm$ 11	$1.8~\pm$ 0.3	$149\pm 8$
WETTZELL	$3.1~\pm$ 0.4	$245~\pm$ 9	$3.0~\pm$ 0.4	$198~\pm$ 6	$1.8~\pm$ 0.4	$119\pm 13$
	$2.2~\pm$ 0.5	$140~\pm$ 12	$0.3~\pm$ 0.3	$66~\pm$ 59	$2.7~\pm$ 0.4	$158\pm 8$

### Mean annual models

The second approach applies mean annual models. Unlike the harmonic model, they are not estimated within a global adjustment but the session-wise residuals of station coordinates are stacked and smoothed within a common year. This procedure was described and applied by Tesmer et al. (2009). In a first step we computed the session-wise station coordinates with respect to the new VieTRF13b reference frame. The parameterisation of the analysis was identical to the approach described in Sect. 2. Then we removed the weighted mean value for each year from the time series to account for possible inter-annual variations. The estimates in the local coordinate system from such modified time series were stacked into one common (mean) year, and we finally applied a smoothing approach with the formal errors of the estimated coordinates as weights.

**Fig. 2:**
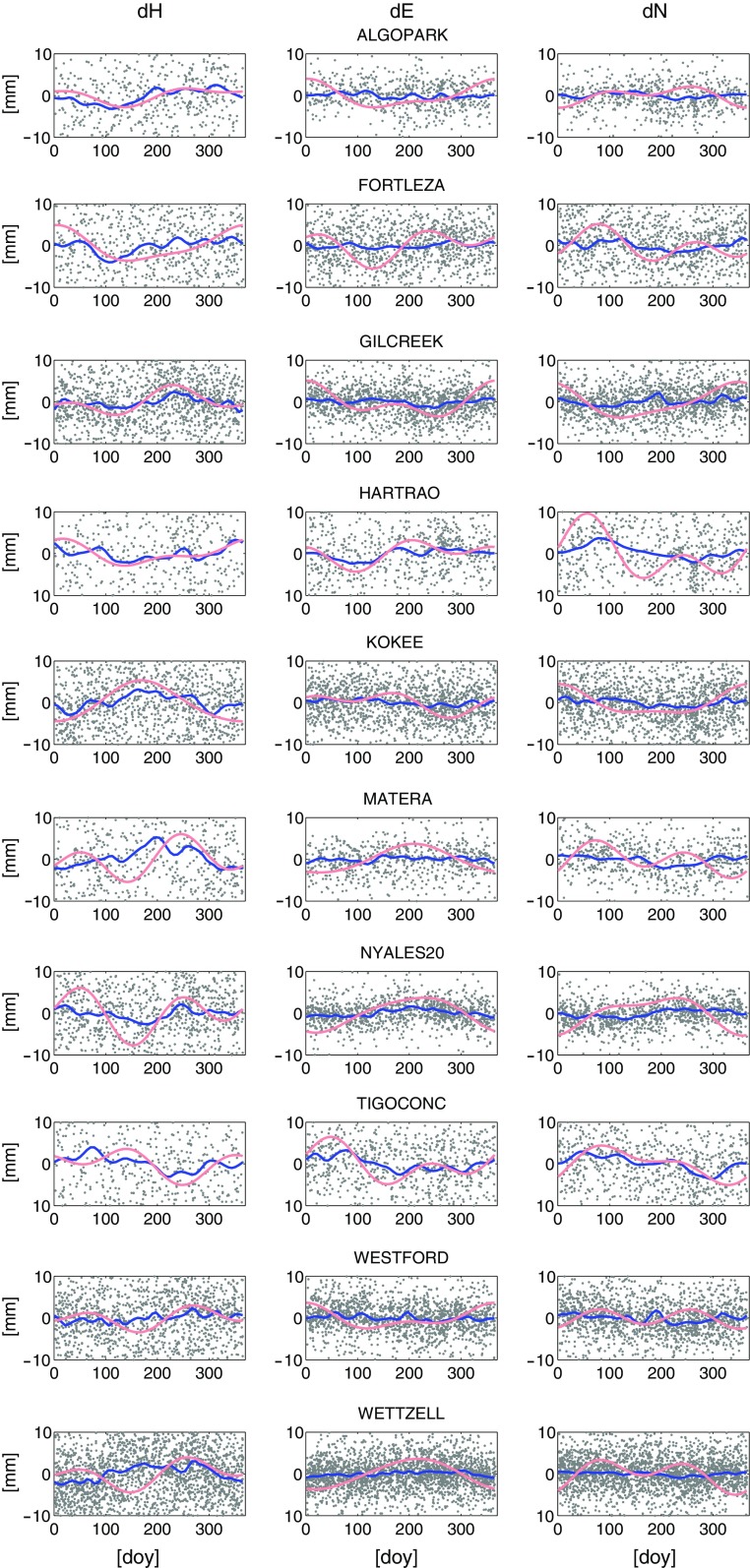
Harmonic (*light red*) and mean annual (*blue*) model at ten most frequently observed stations for height ($d_\mathrm{H}$) and horizontal ($d_\mathrm{E}, d_\mathrm{N}$) coordinate components during a year. The *grey dots* illustrate the coordinate estimates with respect to VieTRF13b without applying any of the two seasonal models

Figure 2 illustrates both models during one year for the ten most frequently observed stations in the data. In light red the harmonic models as sum of annual and semi-annual signals are shown and in blue the averaged mean annual models. The dots depict the coordinate residuals stacked into the year with respect to the VieTRF13b estimated without applying any of the two seasonal models. The left column shows the height component where both models follow the same pattern at most stations. The largest discrepancy can be seen in the height component at station Ny-Ålesund where the harmonic model has a clearly larger amplitude than the mean model. Nonetheless, the harmonic model agrees nicely with the harmonic annual signal in height as estimated for Ny-Ålesund by Tesmer et al. (2009) who determined an annual amplitude of nearly 5 mm from GPS measurements. In the horizontal plane (middle and right columns) the amplitudes of the harmonic model are generally larger than the maxima and minima of the mean annual model. This can be on the one hand caused by the fact that parts of the seasonal variation have already been absorbed by the session-wise datum condition (NNR/NNT) yielding smaller signals in the mean annual models (Böhm et al. 2009). On the other hand, the estimation of the harmonic amplitudes in the global adjustment is more sensitive to the large scatter of the position estimates of the stations and the number of sessions in which the particular station participate. We recognise that further research focusing on the propagation of seasonal signals into the harmonic amplitudes during a global adjustment is needed, especially by a detailed investigation on the application of constraints.

### Validation of the empirical models

**Fig. 3:**
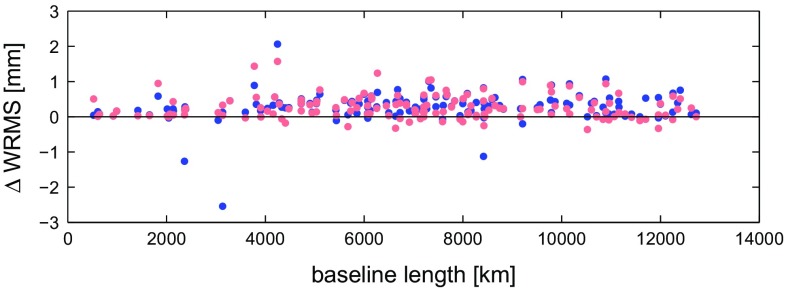
Difference in the baseline length repeatability between the reference solution with omitted seasonal displacement and solution with reduced harmonic signal (S1–S2) plotted as *light red dots*, and solution with the applied mean annual model (S1–S3) shown as *blue dots*

In order to validate our models for seasonal station motion we run two further analysis of the VLBI data. The solutions differ only in the treatment of the seasonal signal in the station coordinates. The first solution (S1) is the reference one where we omitted the seasonal displacement and the parameterisation follows the solution described in Sect. 2. In the second solution (S2) we reduced the harmonic model from the station coordinates a priori, and in the third solution (S3) we modelled the seasonal displacement with the mean annual model. Figure 3 shows the differences in the baseline length repeatability computed as a weighted root mean square deviation (WRMS) for baselines which were observed in more than 50 sessions. The improvement of the WRMS can be seen on 83 % of the baselines with a mean value of 0.3 mm if the harmonic model was applied a priori (red dots) and on 91 % of the baselines (mean value 0.3 mm) if the seasonal displacement was modelled with the mean annual model (blue dots).

## Comparison with hydrology loading and GRACE

The seasonal effect in the station coordinates is supposed to be induced mainly by hydrology. Therefore we compare our models to two series describing hydrology loading displacements. One set (HL1) was computed by the VLBI group at NASA GSFC (Eriksson and MacMillan 2014), the second set (HL2) was provided by Tonie van Dam and Lin Wang from University of Luxembourg. Both are based on the Global Land Data Assimilation System (GLDAS) Noah hydrology model (Rodell et al. 2004) which includes parameters for the soil moisture, snow water equivalent, and canopy water. Furthermore, we compare our models to the displacement derived from GRACE observations (provided by Tonie van Dam and Matthias Weigelt, University of Luxembourg) applying a Gaussian filter of 350 km. For the calculation of GRACE displacements, the Stokes coefficients from the Center for Space Research at University of Texas (CSR) processing centre were used with the C20 coefficients from the CSR solution being replaced with SLR Rel05 estimates from Cheng and Tapley (2004).

**Fig. 4:**
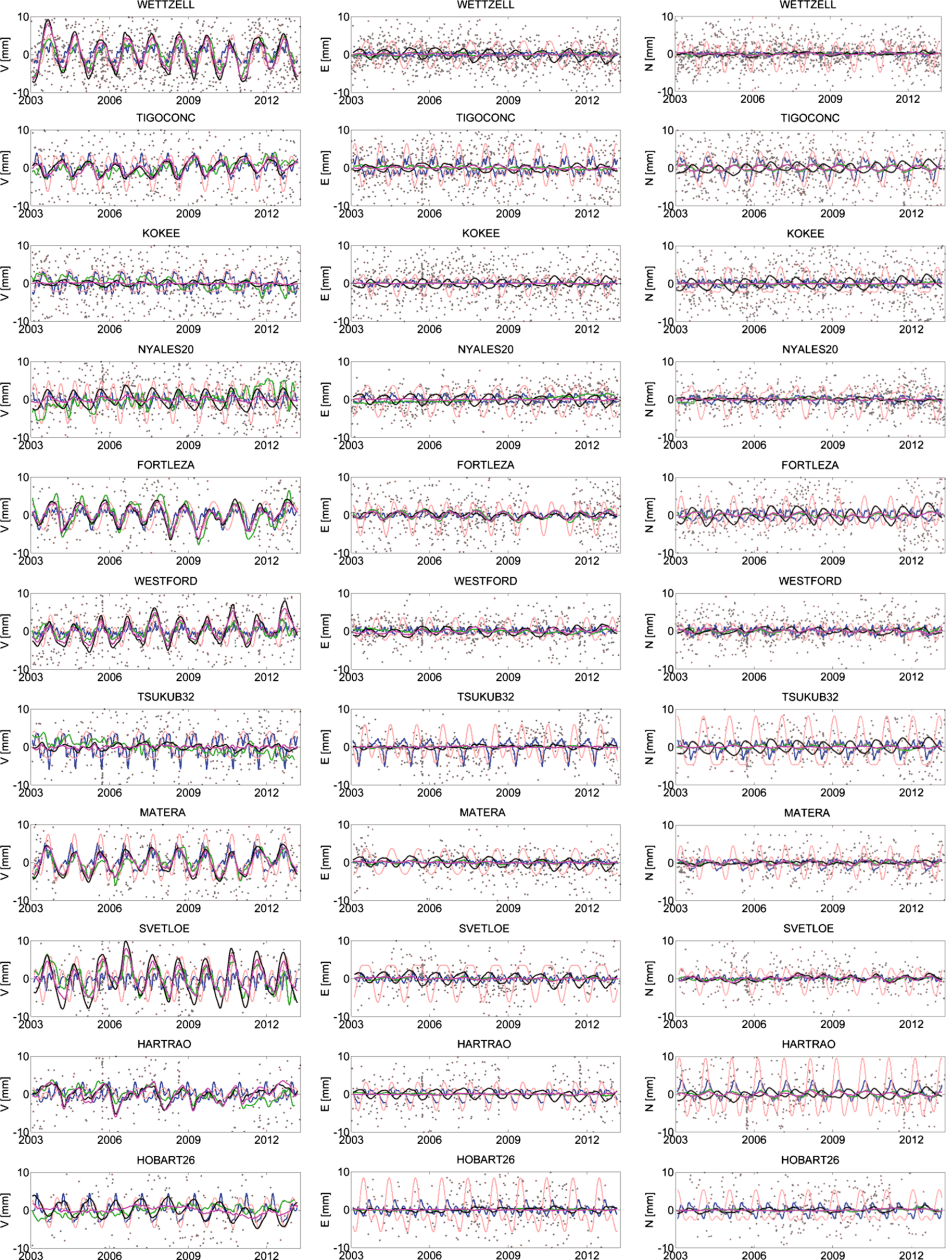
Time series of the height (*left column*), east (*middle column*) and north (*right column*) components at the most frequently observed telescopes from 2003.0 to 2013.3 plotted as *grey dots* without correcting for seasonal effects (shown in descending order according to the number of VLBI sessions). In *light red *the harmonic models and in *blue* the mean annual models are plotted. In *black* and *magenta* the hydrology loading series from GSFC and University of Luxembourg, respectively, are shown. The *green line* depicts the displacement series derived from GRACE

We removed the mean value over 2003.0–2013.3 from each displacement time series derived from the hydrology model and GRACE for a better comparison with our models. The position estimates in the local system of eleven stations with most observations during 2003.0–2013.3 involved in more than 300 sessions are plotted in Fig. 4 as grey dots. The harmonic model at annual and semi-annual periods is shown in light red colour and the mean annual model in blue colour. The hydrology loading displacement time series provided by GSFC (Eriksson and MacMillan 2014) is plotted as a black line and the time series provided by University of Luxembourg in magenta. The green line depicts the surface displacement derived from GRACE. We computed the correlation coefficients between our models and the displacement series based on time series with a 10-day resolution as realized by a Lagrange interpolation of the original displacement series. The correlation coefficients for the height component for stations with more than 100 observing VLBI sessions in that time period are summarized in Table 5.

**Table 5:** Correlation coefficients for the height component at most frequently observed stations involved in more than 100 VLBI sessions during 2003.0 to 2013.3 between our two empirical models and the hydrology loading HL1 (columns two and three), the hydrology loading HL2 (columns four and five), and the displacement derived from GRACE (columns six and seven)

Station	HL1 (GSFC)		HL2 (University of Luxembourg)		GRACE	
	Harmonic	Mean annual	Harmonic	Mean annual	Harmonic	Mean annual
ALGOPARK	0.58	0.77	0.55	0.74	0.46	0.63
BADARY	$-0.39$	$-0.39$	$-0.15$	$-0.26$	0.06	$-0.15$
FORTLEZA	0.43	0.71	0.46	0.69	0.66	0.65
GILCREEK	0.76	0.54	0.55	0.41	0.86	0.68
HARTRAO	$-0.22$	$-0.09$	$-0.25$	$-0.11$	$-0.20$	$-0.09$
HOBART12	0.60	0.00	0.04	$-0.29$	$-0.18$	$-0.23$
HOBART26	0.66	0.70	0.20	0.16	0.07	$-0.03$
KOKEE	$-0.15$	$-0.33$	0.23	0.03	0.32	0.11
MATERA	0.69	0.67	0.69	0.63	0.57	0.51
MEDICINA	0.84	0.45	0.84	0.42	0.75	0.36
NYALES20	0.18	$-0.10$	0.43	0.15	0.39	0.32
ONSALA60	0.22	0.34	0.23	0.32	0.16	0.30
SESHAN25	0.26	0.25	0.38	0.38	0.29	0.30
SVETLOE	0.14	0.09	0.27	0.07	0.25	0.11
TIGOCONC	0.73	0.64	0.74	0.71	0.32	0.22
TSUKUB32	0.31	0.19	0.00	$-0.08$	0.02	$-0.03$
WESTFORD	0.40	0.58	0.34	0.54	0.27	0.52
WETTZELL	0.73	0.75	0.72	0.72	0.66	0.65
ZELENCHK	$$-0.48$$	$$-0.55$$	$$-0.43$$	$$-0.57$$	$$ -0.21$$	$$-0.50$$

Similar comparisons were done, e.g. by Tesmer et al. (2009) who estimated the annual deformation signal from VLBI and GPS, by Tesmer et al. (2011) who compared the height deformation from GPS long-term series with deformations from GRACE, or by Eriksson and MacMillan (2014) comparing their hydrology loading series with VLBI data and GRACE corrections. Our study generally confirms their results. In Table 5 it can be seen that there is a high correlation between the annual model and the hydrology loading and GRACE loading at inland stations, such as Wettzell, Gilcreek or Fortaleza where the hydrology loading is the main contributor to the omitted annual height station displacement in the analysis. At stations where the hydrology loading is low and has strong interannual variations (mainly island and coastal stations like Kokee, Tsukuba, Ny-Ålesund) the correlation is low. The correlation with the hydrology loading series HL1 and HL2 is similar for all stations with the exception of Hobart26. A high correlation coefficient between our seasonal models (harmonic and mean annual) at Hobart26 is obtained with the HL1 (0.66 and 0.70), but a low correlation (0.20 and 0.16) with the HL2. A similarly low correlation is obtained for Hobart26 with the GRACE series (0.07 and $-0.03$). The negative sign means that the hydrology loading does not contribute to the seasonal surface deformation with an annual pattern. At the newly built stations which started their observations after 2003.0, such as Zelenchukskaya, Badary or Hobart12, a low correlation between our models and the compared time series is found which is most probably caused by the short time span of VLBI measurements.

## Influence on the CRF

**Fig. 5:**
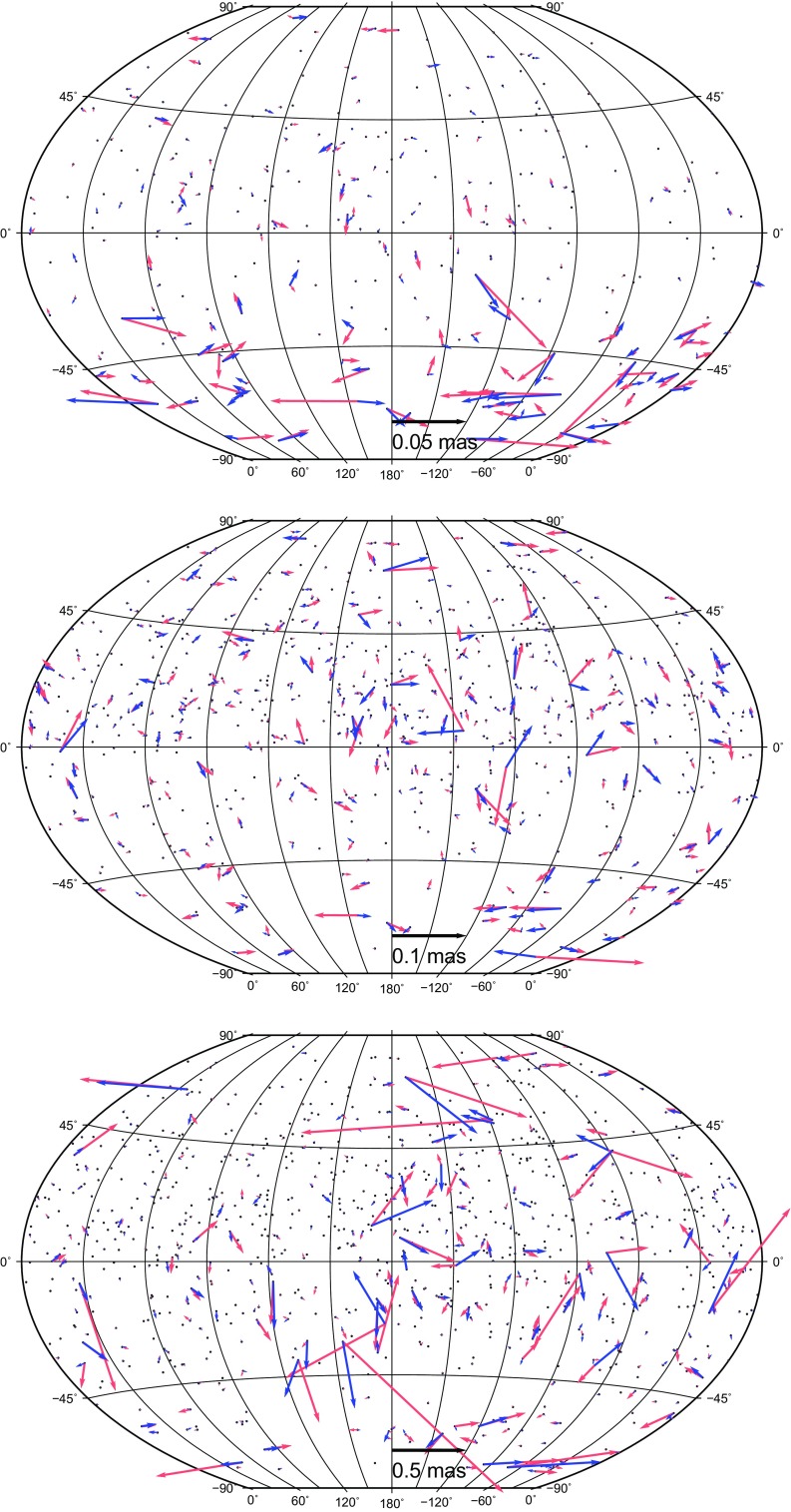
Differences in the estimated celestial frames. *Red arrows* depict the difference S2–S1, *blue arrows* S3–S1. The *upper plot* shows the differences at datum radio sources only, the *middle plot* depicts sources with more than 20 observations in at least two sessions and the *lower plot* includes all radio sources. Note the different scale on the plots

**Fig. 6:**
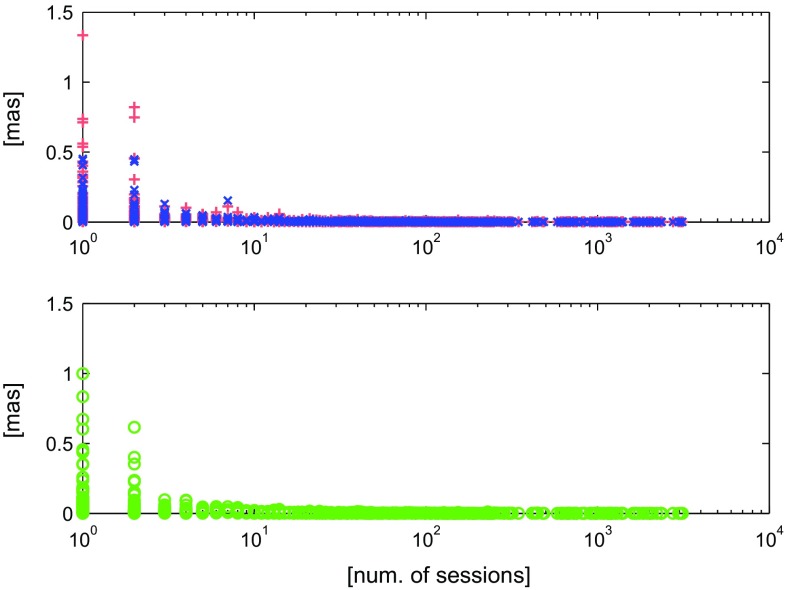
Differences in source positions computed as $\sqrt{((\Delta \mathrm{RA} \cdot \cos \mathrm{De})^2 + \Delta \mathrm{De}^2)}$ w.r.t. number of sessions. In the *upper plot* the *red* “$+$” depict the differences S2–S1, *blue* “*x*” S3–S1. The *green circles* in the *lower plot* show differences S2–S3

In order to assess the influence of the neglected seasonal signal in station coordinates on the estimated celestial reference frame we ran three global adjustments of the VLBI data following the parameterisation of solutions S1, S2, and S3 as described in Sect. 3.3 and determined three CRF. The differences in the estimated radio source coordinates in right ascension ($\Delta$RA) and declination ($\Delta$De) are plotted in Fig. 5. The upper plot shows differences only at the 285 datum sources. The largest differences are at sources in the Southern Hemisphere which is due to the lack of observations and their unequal distribution during a year. The middle plot shows sources which were observed more than 20 times in at least two sessions. In this set of sources the differences are below 0.1 mas. The lower plot depicts the differences at all sources. As shown in Fig. 6 the difference in the estimated source position can exceed 0.2 mas for sources which were observed in one or two sessions only consisting of a limited number of stations. There is no systematic effect in the estimated source coordinates when applying the seasonal models on station coordinates. Table 6 summarizes the WRMS computed over the RA and De differences between the solutions S2 (second column) and S3 (third column) with respect to solution S1. All estimated WRMS values are below one microarcsecond. Table 7 contains weighted rotational parameters between all three estimated CRF. All angles (A1, A2, and A3) are within their formal errors.

**Table 6:** WRMS over the differences for the RA and De component w.r.t. S1; only sources with $m_{\mathrm{RADe}}<1$ mas

Parameter	S2 w.r.t. S1	S3 w.r.t. S1
$\Delta$RA ($\upmu$as)	0.80	0.60
$\Delta$De ($\upmu$as)	0.70	0.49

**Table 7:** Weighted rotational parameters; only sources with $m_{\mathrm{RADe}}< 1$ mas

Parameter	S2–S1	S3–S1	S3–S2
A1 ($\upmu$as)	$0.11~\pm$ 0.12	$0.04~\pm$ 0.08	$-0.07~\pm$ 0.10
A2 ($\upmu$as)	$-0.07~\pm$ 0.12	$0.01~\pm$ 0.08	$0.08~\pm$ 0.10
A3 ($\upmu$as)	$-0.02~\pm$ 0.12	$-0.00~\pm$ 0.08	$0.02~\pm$ 0.10

**Fig. 7:**
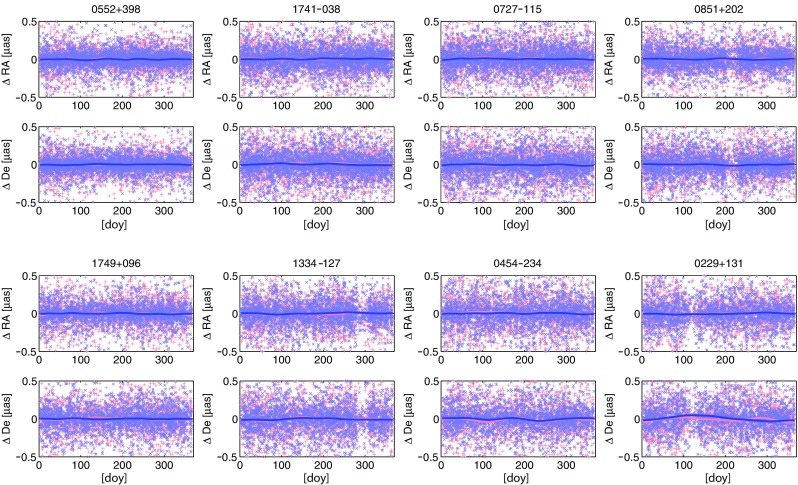
Time series of differences in source coordinates of the eight most observed sources. Plotted are the right ascension and declination from solution S2 (*red* “$+$”) and S3 (*blue* “*x*”) with respect to the solution S1. The *lines* depict the smoothed averaged annual signal

Furthermore, we investigated the effect of the seasonal signal on the time series of estimated source positions. The eight most observed sources were reduced from the session-wise normal equation matrices and estimated as so-called arc-parameters once per session. Figure 7 illustrates the differences in right ascension (upper plots) and declination (lower plots) from solutions S2 (red “$+$”) and S3 (blue “x”) with respect to solution S1 plotted over a common year. The lines depict the smoothed averaged annual signal. The differences in the source coordinates caused by the omitted seasonal signal in the station coordinates are at the sub-microarcsecond range and do not yield any systematic pattern to the frequently observed sources.

## Influence on EOP

**Fig. 8:**
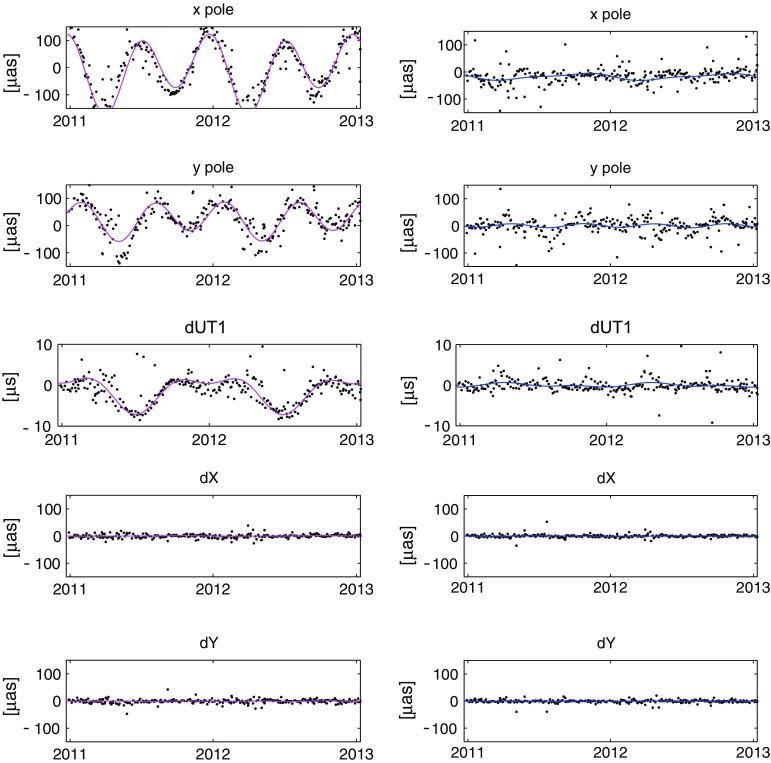
The first column shows the EOP differences from real VLBI observations between solutions S2 and S1, the second column between solutions S3 and S1

Earth orientation parameters determined in solutions S2 and S3 with respect to solution S1 (described in Sect. 3.3) were compared to each other. Plots in the first column of Fig. 8 show the EOP differences (*x*-pole, *y*-pole, $d_\mathrm{UT1}$, $d_X$, and $d_Y$) over 2 years (2011.0–2013.0) between S2 and S1, the second column between S3 and S1. We fitted a model over the whole time series (1984.0–2013.3) comprising an offset, linear trend, and annual and semi-annual harmonics. The model parameters for each EOP difference are summarized in Table 8. Harmonic signals from station coordinates propagate directly into the Earth rotation parameters (ERP; *x*-pole, *y*-pole, $d_\mathrm{UT1}$) with amplitudes of several tens of microarcseconds. Also a large linear drift in *y*-pole (54.6 $\upmu$as/30 years) and $d_\mathrm{UT1}$ ($-3.0~\upmu$s/30 years) are obtained. These systematic differences do not appear in the ERP when applying the mean annual model on station coordinates, which is due to the smaller signals and the inhomogeneous distribution of phases at stations placed in the same regions compared to the harmonic model.

**Table 8:** Parameters of the best-fit model to the EOP time series derived from the VLBI data over 1984.0–2013.3

	*x*-pole	*y*-pole	$d_\mathrm{UT1}$	$d_{X}$	$d_{Y}$
S2–S1					
Offset [$\upmu$(a)s]	$-3.00~\pm$ 0.90	$1.31~\pm$ 0.79	$-0.26~\pm$ 0.04	$0.19~\pm$ 0.09	$-0.16~\pm$ 0.08
Linear trend [$\upmu$(a)s/year]	$-0.35~\pm$ 0.12	$1.82~\pm$ 0.11	$-0.10~\pm$ 0.01	$0.02~\pm$ 0.01	$-0.02~\pm$ 0.01
Annual amplitude [$\upmu$(a)s]	$50.76\pm 1.12$	$19.18~\pm$ 1.02	$3.80~\pm$ 0.05	$0.82~\pm$ 0.12	$0.27~\pm$ 0.11
Semi-annual amplitude [$\upmu$(a)s]	$115.30~\pm$ 1.15	$61.22~\pm$ 1.02	$1.97~\pm$ 0.05	$0.31~\pm$ 0.12	$0.77~\pm$ 0.11
S3–S1					
Offset [$\upmu$(a)s]	$-7.93~\pm$ 1.26	$4.38~\pm$ 1.00	$0.11~\pm$ 0.04	$0.18~\pm$ 0.08	$-0.19~\pm$ 0.08
Linear trend [$\upmu$(a)s/year]	$-0.81~\pm$ 0.16	$-0.30~\pm$ 0.13	$-0.01~\pm$ 0.01	$0.00~\pm$ 0.01	$-0.01~\pm$ 0.01
Annual amplitude [$\upmu$(a)s]	$11.05\pm 1.53$	$0.93~\pm$ 1.31	$0.39~\pm$ 0.05	$0.64~\pm$ 0.11	$0.24~\pm$ 0.11
Semi-annual amplitude [$\upmu$(a)s]	$4.20\pm 1.56$	$7.01~\pm$ 1.28	$0.29~\pm$ 0.05	$0.26~\pm$ 0.11	$0.49~\pm$ 0.11

The reference epoch was set to 1st January 2000

**Fig. 9:**
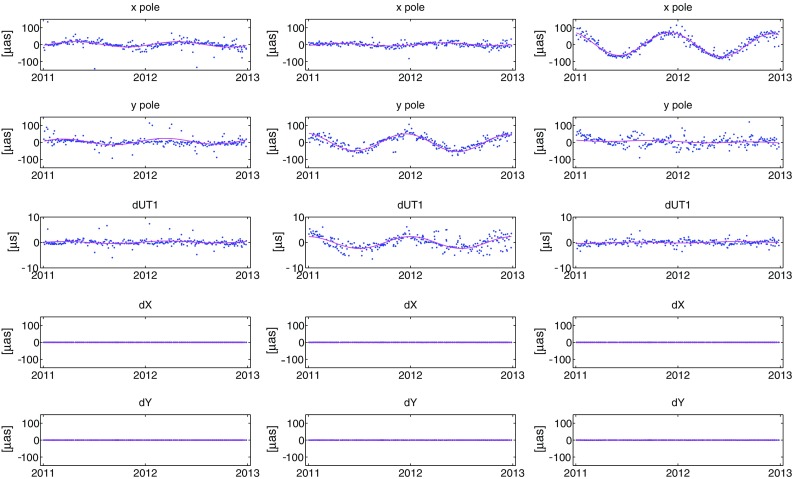
EOP differences estimated from artificial observations. A harmonic signal with an amplitude of 3 mm was added only into the height component (*first column*), only into the east component (*second column*), and only into the north component (*third column*) of the station position

To investigate the propagation of the harmonic signals into the EOP in detail, we created artificial VLBI measurements over 2 years (2011.0–2013.0) based on the real schedules. The corresponding observation files were filled with simulated measurements where the observed time delay is equal to the computed time delay which comes from the models in the analysis software. Analysis of such files provides zero correction of estimated parameters. Based on that approach, we ran three further analyses. In each of them we added a harmonic annual signal with amplitude of 3 mm into only one component of the station position (height, east or north). The phase of the signal at the stations was taken from the real empirical harmonic model determined in Sect. 3.1. Similar to the real observations an offset, linear trend, and annual harmonic were fitted to the EOP estimates (see Table 9). The first row in Fig. 9 depicts the estimates of *x*-pole from these three analyses. The strongest propagation of the harmonic signal into the *x*-pole comes from the north component with an amplitude of $69.50~\pm$ 1.57 $\upmu$as, whereas the largest contribution to the *y*-pole ($51.20~\pm$ 3.20 $\upmu$as) comes from the displacement in east component. Similarly also the $d_\mathrm{UT1}$ is mainly influenced by the east component (with an amplitude of $2.33~\pm$ 0.18 $\upmu$s). The reason for such a separation of the propagation is the geometry of the networks where most of the stations are in Europe or North America. The rows four and five affirm that there is no propagation of harmonic station displacement into the celestial pole offsets $d_X$ and $d_Y$.

**Table 9:** Parameters of the best-fit model to the EOP time series derived from the artificial VLBI observations over 2011.0–2013.0

	*x*-pole	*y*-pole	$d_\mathrm{UT1}$	$d_X$	$d_Y$
Harmonic signal in H					
Offset [$\upmu$(a)s]	$2.09~\pm$ 1.96	$5.28~\pm$ 2.23	$0.07~\pm$ 0.08	$0.00~\pm$ 0.00	$0.00~\pm$ 0.00
Linear trend [$\upmu$(a)s/year]	$-0.36~\pm$ 3.81	$4.10~\pm$ 4.36	$0.20~\pm$ 0.16	$0.00~\pm$ 0.00	$0.00~\pm$ 0.00
Annual amplitude [$\upmu$(a)s]	$15.58~\pm$ 2.87	$17.90~\pm$ 3.32	$0.45~\pm$ 0.12	$0.00~\pm$ 0.00	$0.00~\pm$ 0.00
Harmonic signal in E					
Offset [$\upmu$(a)s]	$1.37~\pm$ 0.76	$-0.91~\pm$ 2.24	$-0.04~\pm$ 0.12	$-0.04~\pm$ 0.01	$-0.06~\pm$ 0.00
Linear trend [$\upmu$(a)s/year]	$2.69~\pm$ 1.13	$-5.90~\pm$ 4.44	$0.01~\pm$ 0.24	$-0.00~\pm$ 0.01	$-0.00~\pm$ 0.01
Annual amplitude [$\upmu$(a)s]	$8.18~\pm$ 1.13	$51.20~\pm$ 3.20	$2.33~\pm$ 0.18	$0.01~\pm$ 0.01	$0.01~\pm$ 0.01
Harmonic signal in N					
Offset [$\upmu$(a)s]	$1.92~\pm$ 1.08	$4.77~\pm$ 1.60	$-0.01~\pm$ 0.06	$0.10~\pm$ 0.01	$-0.03~\pm$ 0.01
Linear trend [$\upmu$(a)s/year]	$-7.62~\pm$ 2.13	$-8.29~\pm$ 3.14	$0.21~\pm$ 0.11	$-0.02~\pm$ 0.01	$0.02~\pm$ 0.01
Annual amplitude [$\upmu$(a)s]	$69.50~\pm$ 1.57	$4.75~\pm$ 2.43	$0.23~\pm$ 0.08	$0.02~\pm$ 0.01	$0.03~\pm$ 0.01

The reference epoch was set to 1st January 2012

## Conclusions

After introducing terrestrial and celestial reference frames (VieTRF13b and VieCRF13b) estimated from VLBI data covering the time span 1984.0–2013.3, we created two kinds of empirical models for the remaining long-period signal in station coordinates, one of them being the harmonic model at annual and semi-annual periods, the second one a non-harmonic mean annual model. We compare them to two sets of hydrology loading corrections and surface displacements derived from GRACE and find good agreement for inland sites frequently observed by VLBI.

Furthermore, the investigations reveal that seasonal station movements do not yield any significant systematic effect on the CRF but can cause a significant change in position of radio sources with small number of sessions non-evenly distributed over the months. On the other hand, we show that harmonic signals in station horizontal coordinates as developed in this work propagate directly into the ERP by several tens of microarcseconds. Future work will deal with a refinement of the harmonic model, in particular with constraints and a reduction of stations for which the horizontal harmonics are estimated, so that we can guarantee a better separation between horizontal station models and ERP.

In any case, we recommend the application of seasonal models a priori on station coordinates in the analysis of VLBI observations.

## Footnotes

1 http://holt.oso.chalmers.se/loading.

2 http://gemini.gsfc.nasa.gov/aplo.
